# Exploring the Pharmacogenetic Landscape: Identification of Clinically Relevant Genotypes by a Nation-Wide Medical Testing Laboratory in Romania

**DOI:** 10.3390/ph18060898

**Published:** 2025-06-16

**Authors:** Cristina Pop, Antoanela Curici, Oliviu Voștinaru, Anamaria Apan, Andra Piciu, Cristina Alina Silaghi, Horatiu Silaghi, Stefan Lucian Popa, Florica Ramona Dorobanțu, Cristina Mogoșan

**Affiliations:** 1 Pharmacology, Physiology and Pathophysiology, Department 2—Pharmacy, Faculty of Pharmacy, “Iuliu Hațieganu” University of Medicine and Pharmacy, 400347 Cluj-Napoca, Romania; pop.cristina@umfcluj.ro (C.P.); oliviu_vostinaru@yahoo.com (O.V.); anamaria.apan@umfcluj.ro (A.A.); cmogosan@umfcluj.ro (C.M.); 2 Morphologic Sciences II, Cellular and Molecular Biology and Histology, Faculty of Medicine, “Carol Davila” University of Medicine and Pharmacy, 050474 Bucharest, Romania; 3 Synevo Laboratories, 077040 Bucharest, Romania; 4 Medical Oncology, Department 10—Oncology, Faculty of Medicine, “Iuliu Hatieganu” University of Medicine and Pharmacy, 400347 Cluj-Napoca, Romania; piciuandra@gmail.com; 5 Endocrinology, Department 5—Medical specialties, Faculty of Medicine, “Iuliu Hatieganu” University of Medicine and Pharmacy, 400347 Cluj-Napoca, Romania; alinasilaghi@yahoo.com; 6 5th Surgery Clinic, Department 6—Surgery, “Iuliu Hatieganu” University of Medicine and Pharmacy, 400347 Cluj-Napoca, Romania; hsilaghi@yahoo.com; 7 2nd Medical Department, “Iuliu Hatieganu” University of Medicine and Pharmacy, 400000 Cluj-Napoca, Romania; popa.stefan@umfcluj.ro; 8 Department of Medical Disciplines, Faculty of Medicine and Pharmacy, University of Oradea, 410087 Oradea, Romania; rdorobantu@uoradea.ro

**Keywords:** pharmacogenetics, personalized medicine, Eastern Europe, Romania genotyping, CYP2D6, CYP2C9, CYP2C19, TPMT, factor V Leiden

## Abstract

**Background:** Pharmacogenetic testing aims to assess the existence of a genetic predisposition that could influence the efficacy or safety of pharmacotherapy. The objective of the present study was to offer a descriptive analysis of the results of the pharmacogenetic tests carried out between 2017 and 2023 within the Synevo laboratories, a provider of medical testing with national expansion. **Method:** To carry out this analysis, data on the following tests offered by the Synevo laboratories were evaluated: CYP2D6, CYP2C9, CYP2C19, TPMT (thiopurine S-methyltransferase), and factor V Leiden. For each type of test, information was collected on the demographics of the patients tested, as well as the genotyping test result. Data were statistically analyzed and interpreted. **Results:** In total, 31.453 pharmacogenetic tests were performed in the considered time interval. Most patients for whom pharmacogenetic testing was performed were women (80%), and as for the age range, patients between 31 and 40 years old (45%) and those between 19 and 30 years old (38%) predominated. In the evaluated sample, genetic variants associated with a potential risk of influencing pharmacotherapy could be identified in a proportion of 56% for the CYP2D6 gene, 41% for the CYP2C9 gene, 52% for the CYP2C19 gene, 12% for the TPMT gene, and 16% for factor V Leiden. **Conclusions:** Pharmacogenetic tests can provide useful information to clinicians in order to personalize pharmacotherapy. Although the interest of medical professionals in these tests is increased, there are currently several impediments to the prescription and routine clinical implementation of pharmacogenetic testing.

## 1. Introduction

Pharmacogenetic testing aims to assess the genetic predisposition that could influence drug treatment. Inter-individual variability in drug response can lead to treatment issues such as the lack of efficacy or the incidence of adverse drug reactions (ADRs). These types of situations are commonly observed in clinical practice and pose a significant burden to patient health and healthcare systems. It is estimated that as much as 50% of the clinical treatments are either inefficient or generate drug-related toxicity [1]. Depending on the context, genetic polymorphisms can explain the variability in drug response in a proportion of 20–95% of cases [2]. This is possible especially because about half of all prescriptions include drugs that could potentially be influenced by genetic variability, and over 95% of the general population carries at least one mutation that is relevant to the drug treatment [3].

Over the decades, a plethora of scientific evidence has accumulated that relates genetic polymorphisms to drug treatment problems [4]. Efforts have been made by medical testing laboratories in implementing genotyping technologies in order to move genetic analysis from the scientific lab, closer to the patient. In this respect, “direct-to-consumer” testing has made an even greater leap, with companies such as 23andMe, who offer FDA-approved pharmacogenetic testing and counseling worldwide [5]. On the other side, organizations such as the Clinical Pharmacogenomics Implementation Consortium (CPIC), the Pharmacogenetics Working Group of the Royal Dutch Association for the Advancement of Pharmacy (DPWG), or the Canadian Pharmacogenomics Network for Drug Safety (CPNDS) have worked on producing and implementing treatment guidelines that could help to tailor drug therapy based on genotype data [6,7]. Thus, after a patient has benefited from genotyping, ideally as part of a preventive pharmacogenetic strategy, the results can be interpreted by a medical professional. Further, recommendations for drug therapy adjustments can be made based on the treatment guidelines available either on the websites of organizations such as CPIC, DPWG, or CPNDS, or by using the database PharmGKB, which has curated such guidelines and made them available in an easy-to-use format [8].

In this context, the European Union has recognized the potential of personalized medicine to enhance disease prevention, diagnosis, and treatment, particularly in the context of an aging population and the rising prevalence of chronic diseases [9]. Legislative frameworks are being developed to facilitate the integration of pharmacogenetic approaches into healthcare systems, with governments aiming to standardize access to genetic testing and treatment options [10]. However, significant disparities exist among member states concerning the implementation of personalized therapy, including variations in healthcare infrastructure, reimbursement policies, and public awareness. These disparities have created an uneven landscape for the accessibility of personalized medical treatments [11]. Moreover, ethical considerations surrounding data privacy and consent, especially concerning genetic information, continue to influence the development of policies aimed at protecting patient rights in the context of personalized therapy [12].

In Romania, Law No. 138 of 24 May 2023, which amends Romania’s Patient Rights Law (no. 46/2003), has a significant impact on the adoption of personalized therapy and pharmacogenetics in the healthcare system [13]. It formally recognizes the rights to individualized treatments tailored to their biological, genetic, or molecular profiles of patients, thus creating a legal foundation for precision medicine [13]. This paves the way for the use of pharmacogenetic testing to optimize drug selection and dosing, reduce adverse reactions, and improve treatment outcomes. Overall, the law marks a crucial step toward integrating advanced, science-driven approaches into routine medical care, ensuring safer and more effective therapies for patients [13].

Given their well-established clinical relevance, our study focused on evaluating the use of key pharmacogenetic markers, such as CYP2D6, CYP2C9, CYP2C19, TPMT, and Factor V Leiden (FVL), which are among the most frequently implicated in drug metabolism variability, adverse drug reactions, and therapeutic efficacy across multiple therapeutic areas.

CYP2C9 is part of the CYP450 superfamily, similar to CYP2D6 and CYP2C19, and is the most abundant CYP2C isoform expressed by the liver, comprising approximately 20% of the total hepatic content. CYP2C9 is also involved in the metabolization of approximately 15% of all clinically used drugs [14]. In addition, because the CYP2C9 gene is highly polymorphic, it is one of the key pharmacogenes involved in the implementation of personalized medicine. Based on CYP2C9 diplotype (allele present on each of the two identical chromosomes), patients can be classified based on CYP2C9 phenotype. Similarly to other genes coding for enzymes, individuals can be categorized into the following CPIC-recommended phenotype categories: poor (PM), intermediate (IM), and normal (NM) metabolizers [15]. Drugs influenced by CYP2C9 polymorphism include oral anticoagulants (acenocoumarol, phenprocoumon, and warfarin); anticonvulsants (phenytoin and fosphenytoin); multiple NSAIDs (e.g., celecoxib, flurbiprofen, lornoxicam, ibuprofen, piroxicam, tenoxicam, and meloxicam); sartans; sulfonylureas (tolbutamide, glimepiride, and gliclazide); and siponimod.

Another CYP450 superfamily member, CYP2C19, plays an important role in the metabolism of approximately 10% of commonly prescribed medications, including proton pump inhibitors like omeprazole, antidepressants such as amitriptyline or sertraline, and the antiplatelet agent clopidogrel [16]. The U.S. Food and Drug Administration (FDA) has included CYP2C19 genotyping information in the drug labels of 28 medicines, recognizing the significance of CYP2C19 genetic variability in drug metabolism and therapeutic efficacy.

Additionally, CYP2D6 plays a critical role in drug metabolism, being responsible for the biotransformation of approximately 20–25% of clinically used medications, including antidepressants, antipsychotics, and opioids [17]. The FDA has included CYP2D6 genotyping information in the drug labels of approximately 78 medicines, and the CPIC has provided guidelines for 26 drugs where CYP2D6 genetic variations are actionable.

TPMT is an important enzyme involved in the metabolism of thiopurine drugs, including azathioprine and mercaptopurine, which are commonly used in the treatment of various conditions, such as leukemia and autoimmune diseases, more specifically mercaptopurine, thioguanine, azathioprine, and cisplatin. TPMT catalyzes the S-methylation of thiopurine compounds, playing a significant role in detoxifying these drugs to mitigate their potential toxic effects on patients [18]. The genetic polymorphism of the TPMT gene has significant implications for drug metabolism, as variations in TPMT activity can lead to different responses to thiopurine therapy. Up to 10% of the population may carry variants associated with reduced enzymatic activity, increasing the risk of severe myelosuppression when exposed to standard drug doses [19]. Notably, more than 20 alleles affecting TPMT function have been identified, highlighting the degree of genetic variability and its importance in tailoring pharmacotherapy [20].

FVL is a well-recognized hereditary thrombophilia risk factor caused by a mutation in the factor V gene, leading to resistance to activated protein C, which normally functions to inhibit coagulation pathways. This condition significantly elevates the risk of venous thromboembolism (VTE), making it a critical consideration in pharmacotherapy, especially for prescribing anticoagulant therapies [21]. The prevalence of the FVL mutation varies by population, being found in about 5% of Caucasians, while it occurs less frequently in African Americans, approximately 1% [22]. Understanding the genetic predisposition to thrombophilia, particularly FVL, is vital for tailoring anticoagulant treatment plans, especially in populations at higher risk, as inadequate management can lead to severe complications such as thrombosis during surgeries or in patients with certain conditions. Consequently, integrating personalized medicine approaches through genetic profiling enhances patient safety and outcomes by informing clinical decision-making tailored to individual genetic risks, emphasizing the importance of pharmacogenetic testing in diverse population settings [23].

The selection of these markers reflects both their inclusion in international pharmacogenetic guidelines (e.g., CPIC and DPWG) and their routine use in personalized therapy decision-making. The objective of this study was to characterize the landscape of pharmacogenetic testing in Romania by conducting a comprehensive descriptive analysis of the frequency and distribution of clinically relevant genotypes identified in tests performed by Synevo laboratories between 2017 and 2023.

## 2. Results

In total, 31,453 pharmacogenetic tests were performed in the considered time interval (2017–2023). Among the tests, FVL is the most requested (31,230 patients), significantly surpassing the other pharmacogenetic tests (CYP2D6, CYP2C9, CYP2C19, or TPMT). Age distribution of patients that underwent pharmacogenetic testing was relatively heterogenous (Table 1). Most patients (83%) were in the 19–40-year-old age interval, more specifically 45% (31–40 years) and 38% (19–30 years), while the other age groups were less well represented (3% for 1–18 years, 16% for 41–50 years, and 11% for over 51 years). Thus, for CYP2C19 genotyping, most patients were in the over-51-years-old interval (66.7%). Additionally, for CYP2D6 genotyping, 66.7% of patients belonged to the 41–50-years group. For TPMT genotyping, most patients were in the two extreme groups, either the 1–18-years group (31.9%) or the over-51-years group (23.2%). Patients that underwent CYP2C9 genotyping were mostly in the 19–30-years group (45.7%) or in the 31–40-years group (39.1%), similar to patients who underwent FVL genotyping, 33.9% and 40.2%, respectively.

To assess whether the distribution of pharmacogenetic tests varied significantly across age groups, we applied both parametric and non-parametric statistical methods. A one-way ANOVA comparing test frequencies across age intervals yielded no significant differences (F = 0.43; *p* = 0.783). Similarly, the Kruskal–Wallis test, a non-parametric alternative suitable for non-normally distributed data, confirmed the absence of statistically significant variation (H = 1.59; *p* = 0.811). Furthermore, marker-specific Kruskal–Wallis tests applied to CYP2C19, CYP2D6, TPMT, CYP2C9, and FVL individually also revealed no significant differences in the distribution of testing across age categories (all *p* ≈ 0.406). These findings suggest that, while absolute test counts vary, no significant age-related trends were observed in the utilization of individual pharmacogenetic tests within the analyzed cohort.

To evaluate potential sex-based differences in the distribution of pharmacogenetic testing, we applied Chi-squared or Fisher’s Exact tests, as appropriate. Statistically significant differences were observed for several markers. Testing for CYP2C19 was more frequently requested in men (63%) than in women (37%) (*p* = 0.0007), while CYP2D6 showed a highly significant predominance in women (94% vs. 6%, *p* < 0.0001, Fisher’s Exact test). Similarly, FVL (factor V Leiden) testing was markedly more frequent in women (80%) than in men (20%) (*p* < 0.0001). In contrast, no statistically significant sex-based differences were identified for TPMT (*p* = 1.00) or CYP2C9 (*p* = 0.53). These findings indicate a non-uniform distribution of certain pharmacogenetic tests between sexes, potentially reflecting differences in clinical indications or referral patterns (Table 2).

The distribution of patients by region in Romania revealed a significantly higher proportion of tests (51%) performed on patients from the Southern region, including the capital (Bucharest). Patients in the eastern region followed (26%), whereas significantly less pharmacogenetic tests were performed in the center of the country (13%) and the western region (10%). The same pattern could be observed upon analysis of each individual pharmacogenetic test (Table 3).

**Figure 1:**
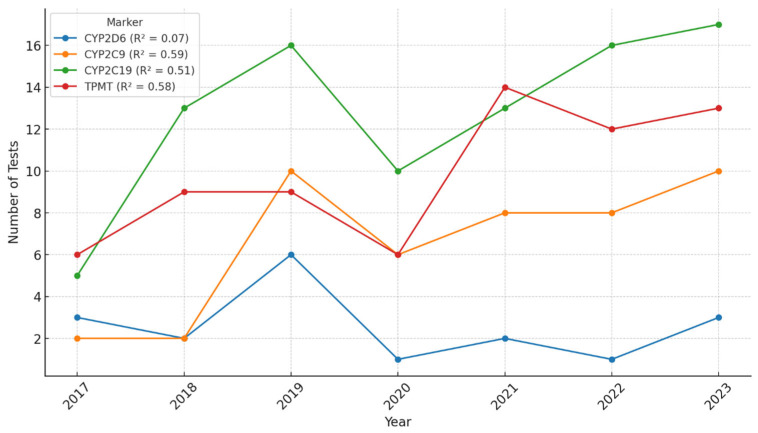
Number of pharmacogenetic tests performed per year, in the interval 2017–2023, at the Synevo laboratories, nation-wide.

Between 2017 and 2023, a steady increase in the number of pharmacogenetic tests performed at Synevo laboratories was observed across all tested markers. Linear regression analyses were applied to each marker to assess the strength of the temporal trend. The coefficient of determination (R^2^) was 0.07 for *CYP2D6*, 0.59 for *CYP2C9*, 0.51 for *CYP2C19*, and 0.58 for *TPMT*, indicating moderate year-over-year growth for *CYP2C9*, *CYP2C19*, and *TPMT*, while the trend for *CYP2D6* was weak. In 2020, we could observe a decrease in the number of tests performed, probably due to the epidemiologic context related to the COVID-19 pandemic. However, the ascending trend continued after 2020, and by 2023, it had reached and even surpassed pre-pandemic levels (Figure 1). These results suggest that although the overall volume of testing increased, the growth trajectory varied by marker, possibly reflecting differences in clinical demand, awareness, or guideline implementation.

**Figure 2:**
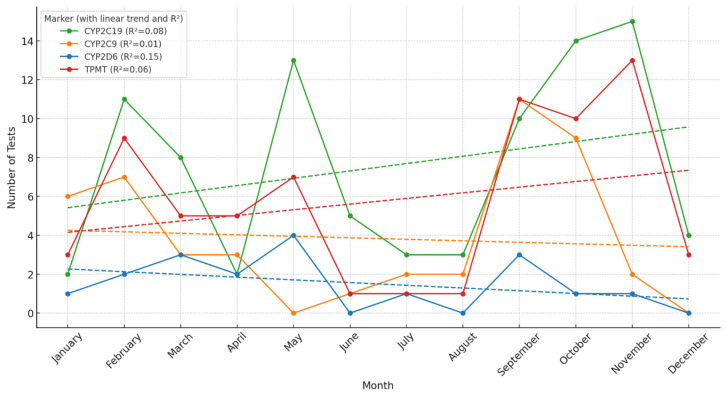
Number of pharmacogenetic tests performed per month, in the interval 2017–2023, at the Synevo laboratories, nation-wide. The dotted lines represent the linear trend for the evolution of pharmacogenetic testing demand (green line CYP2C19, orange line CYP2C9, blue line CYP2D6, red line TPMT).

The monthly distribution of pharmacogenetic testing revealed distinct trends across the analyzed markers. *CYP2C19* demonstrated a consistent upward trajectory throughout the year, with a coefficient of determination (R^2^) of 0.62, indicating a moderately strong linear increase. *TPMT* and *CYP2C9* also showed upward trends, with R^2^ values of 0.55 and 0.46, respectively, suggesting moderate correlations between testing volume and calendar month. In contrast, *CYP2D6* exhibited a relatively flat pattern over the year, with a low R^2^ of 0.14, indicating minimal temporal variation. Overall, we could observe an interesting pattern. For most tests, there were two periods of higher demand of tests, during January–May and during September–November. During summer months, pharmacogenetic testing was lower. Also, in April, we could observe a decrease in pharmacogenetic testing, probably correlated to Easter holidays (Figure 2).

**Figure 3:**
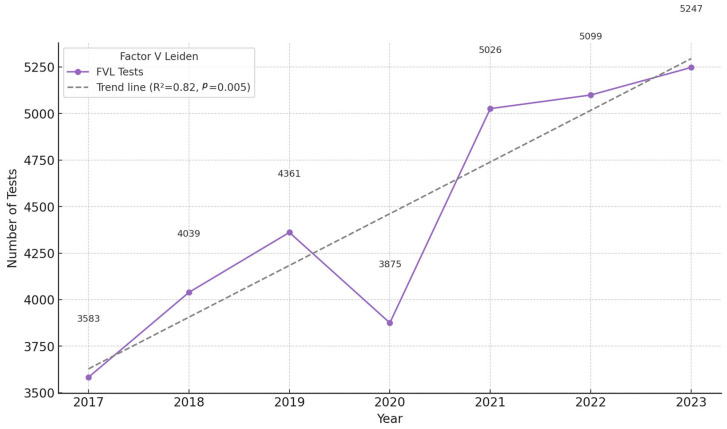
Number of factor V Leiden tests performed per year, in the interval 2017–2023, at the Synevo laboratories, nation-wide.

The factor V Leiden (FVL) genetic test is analyzed separately because of the significantly higher number of tests performed in comparison to the other pharmacogenetic tests (CYP2D6, CYP2C9, CYP2C19, and TPMT). For FVL genotyping, we could also observe an ascending trend over time, with a significantly lower demand for this test in 2020, probably correlated to the COVID-19 pandemic. However, after the pandemic, the demand for FVL increased significantly. Thus, compared to 2017, the number of tests performed in 2023 almost doubled (Figure 3). Linear regression analysis indicated a strong upward trend, with a coefficient of determination (R^2^) of 0.95 and a statistically significant *p*-value of 0.001, suggesting a robust and consistent increase over time. This trend may reflect growing clinical awareness, expanded guideline recommendations, or increased accessibility of thrombophilia screening during the evaluated period.

**Figure 4:**
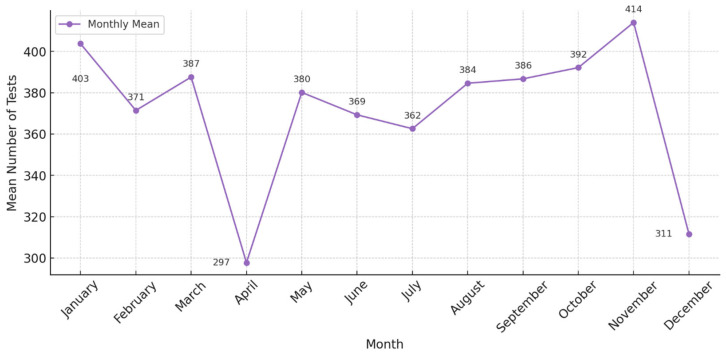
Number of factor V Leiden tests performed per month, in the interval 2017–2023, at the Synevo laboratories, nation-wide.

Analyzing the monthly demand for FVL testing, we could observe a similar pattern compared to the other pharmacogenetic tests. A higher demand of tests in the early months of the year and in autumn (September–November), with a drop in the summer months and in April (Figure 4).

Concerning the genotypes detected, for all pharmacogenetic tests, the wild-type gene, i.e., the normal gene variant, predominated. However, for each test, gene variants that could be of interest concerning pharmacotherapy safety or efficacy could be detected in a proportion of patients.

For phase I drug-metabolizing enzymes, such as CYP isoenzymes (CYP2C9, CYP2C19, and CYP2D6), most patients could fit into the phenotype of normal (rapid) metabolizer, while phenotypes such as intermediate, slow, or ultrarapid metabolizers could also be detected.

The distribution of CYP2C9 genotypes and their corresponding metabolic phenotypes was assessed, and a potential association with patient sex was investigated (Table 4). Genotype–phenotype concordance was observed, with *CYP2C9* *1/*1 corresponding to normal metabolizer status (59%), *1/*2 to intermediate metabolizer (15%), and *2/*3 to poor metabolizer (26%). A Chi-squared test for independence between genotype and sex revealed no statistically significant association (χ^2^ = 0.84, *p* = 0.655), indicating that the distribution of CYP2C9 genotypes was similar between men and women in this cohort.

The distribution of CYP2C19 genotypes and corresponding metabolizer phenotypes was evaluated, including potential sex-based differences. The most frequent genotype was *CYP2C19* *1/*1, corresponding to normal (rapid) metabolizer status (48%). Other observed genotypes included *1/*2 (12%), *2/*17 (10%), *2/*2 (4%), *2/*3 (2%), *1/*17 (17%), and *17/*17 (7%). A Chi-squared test of independence showed no statistically significant association between genotype distribution and sex (χ^2^ = 7.74; *p* = 0.258), suggesting that the frequency of CYP2C19 genotypes is relatively similar in male and female patients (Table 5).

The distribution of CYP2D6 genotypes and associated phenotypes was examined, along with potential differences between sexes (Table 6). The most frequent genotype was the combination of normal allele and mutant null allele (CYP2D6 *1/*5), generating a phenotype of intermediate metabolizer that could be identified in 39% of patients. *CYP2D6* *1/*1 (17%) and *1/*35 (17%), corresponding to normal metabolizer statuses, were also frequent. A smaller proportion of patients could be categorized as slow metabolizers, based on a heterozygous genotype of null allele *4/*15 (11%) or *5/*40 (6%). Despite the observed predominance of female patients in the dataset, a Chi-squared test of independence found no statistically significant association between CYP2D6 genotype and sex (χ^2^ = 5.29; *p* = 0.507).

TPMT is a phase II drug-metabolizing enzyme that methylates drugs and some endogenous substrates. Table 7 shows that most patients undergoing TPMT genotyping (88%) presented the wild-type allele (TPMT *1), while variant alleles *3A and *3B were observed in 6% of cases each. A Chi-squared test for independence indicated no statistically significant association between genotype and sex (χ^2^ = 1.04; *p* = 0.594), suggesting that TPMT genotypes are similarly distributed among male and female patients in this sample.

Finally, most patients in our study underwent genotyping in order to detect the mutant variants for factor V Leiden (rs6025 mutation). No mutation was detected in 84% of patients. The patients presenting with the rs6025 mutation (16%) were either heterozygous (12%), or homozygous (4%) (Table 8). A Chi-squared test revealed a highly significant association between genotype and sex (χ^2^ = 253.44, *p* < 0.0001), indicating a non-uniform distribution. Specifically, the proportion of heterozygous and homozygous variants differed significantly between men and women, suggesting potential sex-related differences in genetic predisposition or test referral patterns related to thrombophilia risk.

## 3. Discussion

In order to evaluate the use of pharmacogenetic testing in Romania, we analyzed the database of Synevo Laboratories. This private company is one of the leading providers of medical laboratory services in Romania, including a network of hundreds of collection centers and multiple high-capacity laboratories, and thus covering all Romanian geographical regions [24].

However, despite the growing interest of Romanian medical professionals in personalized medicine [25] and the efforts made by private laboratories in offering pharmacogenetic testing in Romania, the use of this approach to personalized therapy remains relatively low, highlighting a gap between medical innovation and its practical implementation in clinical settings.

Although the total number of pharmacogenetic tests analyzed in this study is substantial, the number of individuals tested for CYP2C9, CYP2C19, and CYP2D6 appears comparatively small. This reflects current clinical practices in Romania, where CYP genotyping is not yet broadly implemented and is typically reserved for selected therapeutic contexts, such as antidepressant use or antiplatelet therapy. In contrast, testing for TPMT and factor V Leiden is more frequently requested due to well-established clinical indications, such as thiopurine therapy and thrombophilia screening.

The trends of pharmacogenetic-testing utilization in Romania reflect a complex interaction of seasonal health-seeking behaviors, cultural practices, and increased awareness of the importance of pharmacogenetic insights in personalized medicine. Our data show an uneven distribution of pharmacogenetic tests among sexes that may partly stem from inherent biological differences. For example, women often experience more adverse drug reactions and have been found to exhibit different pharmacokinetic patterns for various medications, necessitating greater scrutiny through pharmacogenetic testing [26]. In contrast, the higher prevalence of CYP2C19 testing among men may reflect gender-based differences in the types of medications prescribed or health conditions diagnosed, particularly concerning cardiovascular issues, where men are often at greater risk [27]. Also, the predominance of women in CYP2D6 testing could possibly relate to higher instances of mood disorders in the female population and their awareness of pharmacogenetic factors affecting treatment efficacy [28].

Sociocultural factors also play a significant role in health-seeking behavior, influencing who decides to seek pharmacogenetic testing. Women traditionally engage more with healthcare services and may be more proactive in seeking genetic testing for conditions such as those related to TPMT and CYP2D6, which have implications for emotional health or chronic conditions often treated with psychiatric medications [28].

The regional disparities in the distribution of pharmacogenetic tests in Romania, with a significant portion (51%) being performed in the southern region, including the capital city, can be attributed to several intertwined factors. Firstly, Bucharest benefits from a more developed healthcare infrastructure compared to other areas. Urban centers often have greater access to specialized healthcare services, including genetic testing and personalized medicine initiatives, due to the concentration of healthcare institutions and professionals [29]. Patients in these regions may be more likely to undergo pharmacogenetic testing as a result of increased availability of these services and greater proximity to healthcare facilities offering such tests. Secondly, public awareness and demand for pharmacogenetic testing may be higher in urban areas. Patients in big cities may be more engaged in healthcare due to better access to information and resources that promote understanding of the benefits of pharmacogenetic testing [30]. Thirdly, the demographics of the populations in different regions also play an important role. Younger populations in urban areas may be more proactive in seeking testing, particularly in response to lifestyle-related health issues. The data suggest a higher percentage of younger patients seeking testing, likely motivated by health concerns or family histories of disease [31]. Conversely, areas that have older populations present fewer requests for pharmacogenetic tests. Addressing these disparities is crucial for the equitable implementation of pharmacogenetics across all regions.

Regarding the evolution of pharmacogenetic testing in time, we could see an ascending trend, interrupted by the COVID-19 pandemic. While the initial decrease in pharmacogenetic testing during the COVID-19 pandemic can be explained by restricted healthcare access and patient hesitance, the subsequent recovery and rise in testing volumes are attributed to heightened awareness, healthcare system adaptation, and evolving clinical practices that prioritize personalized medicine [30].

Concerning pharmacogenetic testing patterns within one year, the surge in testing during the first half of the year (January–May) is likely influenced by New Year’s resolutions and the heightened awareness of health and wellness that often accompanies the start of a new year. Individuals are more likely to engage in health checks and preventative healthcare practices immediately after the holiday season. This trend aligns with the findings from previous studies, which indicate that people often prioritize health and medical interventions at the beginning of the year [32]. The notable decrease in the month of April, likely correlated with the Easter holidays, reflects the impact of cultural and religious observances on healthcare-seeking behavior. During significant holidays, clinics may have reduced hours or limited staffing, affecting the availability of testing services. Moreover, individuals may prioritize family gatherings and celebrations over medical appointments during these periods. Such patterns are supported by studies that examine healthcare activity fluctuations around major holidays, where patients may defer non-urgent testing in favor of festivities [33].

In recent years, increasing awareness of pharmacogenetic testing and its implications for personalized medicine has contributed to heightened interest and demand for testing resources [34]. Initiatives highlighting the importance of genetic testing in optimizing drug therapies may play a role, especially in the months following public health campaigns that promote awareness and education [25]. This growing knowledge encourages patients to seek pharmacogenetic testing more actively during periods when they engage with healthcare professionals [34].

### 3.1. CYP2C9 Pharmacogenetic Testing

In our study, we have found a higher frequency of CYP2C9 *2 and CYP2C9 *3 alleles (41% of patients), as compared to other studies [35]. One possible explanation could be that we did not sample the general population, but our sample included patients that had an indication for pharmacogenetic testing based on a presumption that they may be positive for actionable pharmacogenes. Thus, it would be expected that these patients present with a higher frequency of alleles that possibly modify pharmacokinetics and pharmacodynamics of drugs. From the functionally relevant CYP2C9 alleles, *2 and *3 were the most abundant in Europe and the Middle East. Additionally, CYP2C9 *3 was also common in South Asia, as well as in some countries in South America, such as Uruguay, Columbia, and Brazil [35]. In vitro, CYP2C9 *2 reduces enzyme activity by 50–70%, whereas CYP2C9 *3 almost completely abrogates enzyme function (reduction of 75–99%) [35].

In our population, around 15% of the patients presented with a phenotype of intermediate metabolizer (CYP2C9 *1/*2), and 26% were poor metabolizers (CYP2C9 *2/*3). This translation of genetic variability into functional phenotypes can reveal that a great proportion of patients, around 40%, can benefit from therapy adjustment for drugs metabolized by CYP2C9, most specifically for drugs with a narrow therapeutic index.

Due to the significant impact of CYP2C9 variations, the US Food and Drug Administration (FDA) and the European Medicines Agency (EMA) include CYP2C9 genotyping in the drug labels or summary of product characteristics of 19 drugs. Specifically, testing is required for the sphingosine-1-phosphate receptor modulator siponimod in multiple sclerosis, and CYP2C9 genotype is also considered actionable information for dosage of warfarin, phenytoin, and several non-steroidal anti-inflammatory drugs (NSAIDs) [35].

The most common clinical application of CYP2C9 genotype in formation described to date is its use, together with VKORC1 and possibly CYP4F2, to guide warfarin dosing. Individuals with one or two decreased or no function alleles have decreased metabolism of the more potent S- enantiomer of warfarin and increased risk of bleeding with usual warfarin doses (i.e., 5 mg per day), and thus, a lower warfarin dose is required to achieve therapeutic anticoagulation. Three multisite clinical trials have examined the efficacy of CYP2C9 plus VKORC1 genotype-guided warfarin, of which two trials demonstrated favorable effects of a genotype-guided approach on the outcome of improved anticoagulation control or reduction in risk for bleeding, thromboembolism, death, or supratherapeutic anticoagulation following total joint arthroplasty.

CYP2C9 decreased, and no function alleles similarly lead to increased exposure to other CYP2C9 substrates, which may increase the risk for serious adverse effects, including neurotoxicity with phenytoin, gastrointestinal bleeding and adverse cardiovascular effects with NSAIDs, and bradycardia with siponimod. Siponimod is contraindicated for poor metabolizers (PMs) with the CYP2C9 *3/*3 genotype that are expected to have little-to-no enzyme activity. Thus, genotyping is required prior to siponimod initiation. Lower phenytoin maintenance doses are recommended for patients with genotypes associated with significant reductions in enzyme activity. For NSAIDs, the consequences of decreased CYP2C9-mediated metabolism are expected to be greatest for drugs with a long elimination half-life (e.g., piroxicam, tenoxicam, and meloxicam) and less significant for NSAIDs with shorter half-lives (e.g., celecoxib and ibuprofen). This is reflected by CPIC guidelines recommending that in PMs (e.g., CYP2C9 *2/*3 or *3/*3 genotypes), piroxicam, tenoxicam, and meloxicam be avoided, whereas celecoxib and ibuprofen may be used but should be started at lower-than-usual doses.

Sex-related differences in CYP2C9 expression have been investigated to a limited extent, and to date, there is no evidence supporting meaningful differences between males and females.

One study found slower metabolism of losartan in females compared with males [36]. However, considering that estrogens upregulate CYP2C9 expression via the estrogen receptor, studies on sex differences should consider this possible confounder [37]. Also, it seems that age could influence CYP2C9 expression, with CYP2C9 levels lower in children and increasing linearly up to 20 years [38]. Findings for the effect of age on CYP2C9 contrast with CYP2C19, where levels appear to be higher in children than in adults.

### 3.2. CYP2C19 Pharmacogenetic Testing

Our results show that for CYP2C19, generally more genotypes could be detected compared to CYP2C9. CYP2C19 is known to exhibit a higher degree of polymorphism compared to CYP2C9, which may be linked to evolutionary pressures and the historical genetic landscape of various populations, including Romanians. This higher variability allows for a broader range of metabolizer phenotypes, making CYP2C19 particularly relevant in clinical contexts, especially concerning drug metabolism and patient response to medications [39].

Also, the clinical implications associated with CYP2C19 polymorphisms, particularly regarding drugs such as clopidogrel and proton pump inhibitors (PPIs), likely increase the testing rates among healthcare practitioners who are aware of its relevance in treatment efficacy and safety. This is evidenced in guidelines that recommend genotype testing to optimize therapy with these medications, especially in populations where such medications are frequently prescribed [40].

In our study, most patients who were tested for CYP2C19 variability were over 51 years old; thus, they were most likely under chronic treatment with drugs possibly influenced by CYP2C19 metabolism. Older adults are indeed more likely to take multiple medications, a phenomenon known as polypharmacy. For instance, one study indicated that approximately 26.3% of older adults in Taiwan were prescribed five to nine medications daily, while 15% used ten or more drugs [41]. In older populations, where polypharmacy is prevalent due to multiple comorbidities, the implications of CYP2C19 variability become even more critical. Poor metabolizers may experience increased drug concentrations and adverse effects when prescribed medications that rely on CYP2C19 for metabolism, such as clopidogrel [42]. Studies indicate that genetic polymorphisms in CYP2C19 not only influence treatment efficacy but also increase the likelihood of hospitalizations among patients experiencing adverse drug events due to polypharmacy [43].

The prevalence of the CYP2C19 *17 haplotype associated with ultrarapid metabolism of CYP2C19 across the evaluated population has important implications. A growing body of research suggests that the distribution and frequency of alleles, including CYP2C19 *17, may be influenced by factors such as demographic history, ethnicity, and geographic region [44]. In Romania, the prevalence of CYP2C19 ultrarapid metabolizers can be explained by genetic drift, population admixture, and historical migrations that have shaped the genetic landscape of the region, positioning it similarly to various Eastern and Central European populations in terms of genetic diversity and overall ancestry. Various studies have indicated that populations from Eastern Europe, including Romania, exhibit a wide range of CYP2C19 genetic variability, encompassing both normal and ultrarapid metabolizer phenotypes [44]. The high prevalence of the *17 allele may influence drug responses and treatment outcomes for medications metabolized by CYP2C19, such as clopidogrel and certain antidepressants, leading to differential therapeutic effectiveness and risks of adverse drug reactions when compared to other populations with lower frequencies of this variant [44]. Additionally, clinical guidelines suggest that understanding the frequencies of these haplotypes will aid in the implementation of personalized medicine practices in Romania, optimizing drug prescriptions based on genetic testing results [44]. As healthcare systems increasingly adopt pharmacogenetic approaches, the high prevalence of ultrarapid metabolizers in the Romanian population highlights the urgency for tailored drug therapies that consider these genetic differences. This proactive approach could streamline treatment protocols, thus mitigate the risks of therapeutic failure and enhancing overall patient outcomes [45]. Moreover, the implementation of clinical decision support systems that incorporate CYP2C19 genotype data could facilitate genotype-guided prescribing, benefiting individuals identified as ultrarapid metabolizers [46]. Such measures would lead to a more effective healthcare model that aligns pharmacotherapy with individual genetic profiles, particularly as Romania continues to develop its public health strategies around pharmacogenomics [30].

### 3.3. CYP2D6 Pharmacogenetic Testing

The concentrations of CYP2D6 tests in the 41–50 age cohort may reflect a combination of ongoing treatment adjustments and monitoring for mental health disorders, where pharmacogenetic factors become particularly critical in personalized medicine approaches [47].

The findings from the CYP2D6 genotyping study, which revealed that 40% of patients were normal metabolizers, 39% were intermediate metabolizers, and a smaller proportion were classified as slow metabolizers, align with data reported in studies indicating similar allele distributions across various populations. For instance, the prevalence of normal metabolizers typically falls within this range in studies conducted in European cohorts, suggesting that the Romanian population exhibits alleles consistent with a broader ethnic background [48]. The significant percentage of individuals categorized as intermediate metabolizers (39%) is noteworthy and reflects the prevalence observed in populations characterized by diverse genetic backgrounds. For example, studies have documented comparable intermediate metabolizer rates in Middle Eastern and African populations, indicating a potential genetic diversity contributing to these findings [49,50]. The emergence of slow metabolizers, identified in 6% of patients with a heterozygous genotype (CYP2D6 4/15) in this study, is within the range reported in the literature, though some sources highlight varying rates across different ethnic groups [51,52]. Analyzing these findings in the context of Romanian patients suggests a complex interplay of genetic factors that may align with historical migration patterns and regional population genetics. The high variability in CYP2D6 alleles can be attributed to population admixture, where distinct genetic backgrounds may yield a diverse array of phenotypes, impacting drug metabolism and therapeutic response [53,54].

### 3.4. Thiopurine S-Methyltransferase (TPMT) Pharmacogenetic Testing

Our results, indicating that 88% of patients undergoing TPMT genotyping presented the wild-type allele (TPMT *1), are consistent with findings in various populations, highlighting the prevalence of this allele among different ethnic groups. For instance, a study by Ladić et al. reported a similar predominance of the TPMT *1 allele in Croatian patients with inflammatory bowel disease, while also noting *3A as a more common pathological variant allele in that population [55]. This comparison emphasizes the genetic variability of TPMT variants, as research has demonstrated that while mutant alleles (such as TPMT *3A and *3B) exist, their frequency and implications for drug metabolism can differ across populations.

Further studies have found that the distribution of TPMT polymorphisms varies regionally; for example, Almoguera et al. indicated that in Asian populations, *3C is often the most prevalent variant, whereas *3A is more common in Caucasian populations [56]. Additionally, the identification of *3A and *3B alleles in patients categorized as slow metabolizers underscores the clinical importance of understanding these genetic variations, as individuals with these alleles may experience heightened risks of adverse reactions to thiopurine medications due to diminished metabolic capacity [57]. In populations like the Iraqi cohort studied by Kadhum et al., the presence of TPMT variants is associated with significant implications for treatment efficacy and safety, supporting the recommendation that pharmacogenetic testing should be standard practice to reduce adverse drug reactions and optimize dosing among this diverse population [58].

### 3.5. Factor V Leiden (FVL) Pharmacogenetic Testing

Our findings indicate that 16% of patients presented with the FVL mutation (rs6025), with 12% being heterozygous and 4% homozygous. In Caucasian populations, the frequency of the FVL mutation typically ranges from 5% to about 10%, particularly among those at risk for venous thromboembolism (VTE) [59]. Notably, some regions within Europe have reported frequencies as high as 10–15% in the general population [59]. In several European studies, researchers highlighted the FVL mutation as a primary genetic risk factor for thrombosis, reinforcing the clinical necessity for screening in high-risk populations [22]. In contrast, the results from the current analysis suggest a slightly higher incidence of the FVL mutation compared to some studies focusing on specific cancer or thrombosis cases. The observed 16% mutation rate indicates that while the Romanian cohort aligns with global data suggesting that FVL is a significant risk factor for thrombosis, population-specific genetic characteristics or environmental factors may also influence these results. When examining the distribution of FVL among genders, the findings indicate a similar prevalence of the mutation across male and female patients. This observation is consistent with studies suggesting no substantial gender-based differences in the presence of this polymorphism. However, the slight variations in the presence of heterozygous and homozygous mutations reported here underscore the need for localized studies that consider demographic contexts when evaluating genetic risk factors for thrombosis [60].

Moreover, the age distribution observed reflects the prevalence of certain conditions and the health-seeking behavior of different demographics. The predominance of younger adults undergoing FVL testing may indicate greater awareness of thrombosis-related risks, possibly influenced by family histories or personal medical backgrounds, which motivates testing early [61].

## 4. Materials and Methods

This study is a retrospective analysis based on anonymized pharmacogenetic test results extracted from the Synevo laboratories database. No personal identifiers were collected or processed. In accordance with applicable ethical guidelines, the study was conducted based on a scientific collaboration agreement that ensured confidentiality and limited use strictly for scientific purposes.

The analysis focused on the genotyping of key pharmacogenetic markers, specifically CYP2D6, CYP2C9, CYP2C19, TPMT (thiopurine S-methyltransferase), and factor V Leiden. For each pharmacogenetic test, we collected comprehensive demographic data of the patients, as well as the results of the genotyping analyses. These data were subsequently subjected to statistical analysis using SPSS Statistics Version 27.0 (IBM Corp., Armonk, NY, USA) in order to describe the prevalence and distribution of different alleles and phenotypes within the studied population. The findings were compared to other studies in order to understand the genetic landscape of Romanian patients in relation to drug metabolism and therapy personalization.

## 5. Conclusions

Between 2017 and 2023, pharmacogenetic testing in Romania experienced a rise in demand, performing over 31,000 tests, with factor V Leiden (FVL) as the clear leader. The majority of patients were women and adults aged 19 to 40, though each test showed unique demographic patterns: for instance, CYP2C19 testing was more frequent in older adults, while TPMT testing was notably common among children and seniors. Testing rates followed a steady upward trend, briefly interrupted in 2020 due to the COVID-19 pandemic, but quickly recovered, surpassing pre-pandemic levels. Seasonal patterns were evident, with peaks in spring and autumn, and lower activity during summer and around Easter. Genetic analysis revealed that while most patients carried the normal gene variants, a significant number of them harbored mutations relevant to drug metabolism, especially for CYP enzymes. These insights reflect a growing awareness and adoption of pharmacogenetic testing in Romania, paving the way toward safer, more personalized treatments. A key limitation of this study is the lack of access to clinical outcome data, as the analysis was based on anonymized retrospective laboratory records. As a result, we were unable to correlate specific genotypic profiles with therapeutic responses, adverse drug reactions, or clinical decision-making outcomes.

As pharmacogenetics aims to optimize therapeutic efficacy and minimize adverse drug reactions, it empowers patients to receive the most appropriate medications based on their unique genetic makeup. However, although the interest of Romanian medical professionals in these tests is increased, there are currently several impediments to the prescription and routine clinical implementation of pharmacogenetic testing.

## Figures and Tables

**Table 1:** Age distribution of patients requiring pharmacogenetic testing.

Age Interval (Years)	CYP2C19 N (%)	CYP2D6 N (%)	TPMT * N (%)	CYP2C9 N (%)	FVL N (%)
1–18	1 (1.1)	1 (5.6)	22 (31.9)	1 (2.2)	781 (2.5)
19–30	1 (1.1)	2 (11.1)	8 (11.6)	21 (45.7)	10,593 (33.9)
31–40	18 (20)	2 (11.1)	10 (14.5)	18 (39.1)	12,563 (40.2)
41–50	10 (11.1)	12 (66.7)	13 (18.8)	2 (4.3)	4286 (13.7)
51+	60 (66.7)	1 (5.6)	16 (23.2)	4 (8.7)	3007 (13.7)

* TPMT—thiopurine S-methyltransferase; FVL—factor V Leiden.

**Table 2:** Sex distribution of patients requiring pharmacogenetic testing.

Sex	CYP2C19 n (%)	CYP2D6 n (%)	TPMT * n (%)	CYP2C9 n (%)	FVL n (%)
Women	34 (37)	17 (94)	34 (49)	21 (46)	24,923 (80)
Men	58 (63)	1 (6)	35 (51)	25 (54)	6307 (20)

* TPMT—thiopurine S-methyltransferase; FVL—factor V Leiden.

**Table 3:** Distribution of patients requiring pharmacogenetic testing by region in Romania.

Region	CYP2C19 n (%)	CYP2D6 n (%)	TPMT * n (%)	CYP2C9 n (%)	FVL n (%)
Center	5 (6)	2 (11)	6 (9)	10 (22)	3980 (13)
East	11 (12)	4 (22)	5 (7)	21 (46)	8263 (26)
South	63 (70)	8 (44)	49 (71)	11 (24)	15,996 (51)
West	11 (12)	4 (22)	9 (13)	4 (9)	2991 (10)

* TPMT—thiopurine S-methyltransferase; FVL—factor V Leiden.

**Table 4:** Genotype and phenotype distribution for CYP2C9.

Genotype	n (%)	Phenotype	n (%)	Men n (%)	Women n (%)
CYP2C9 *1/*1	27 (59)	Normal (rapid) metabolizer	27 (59)	12 (48)	15 (52)
CYP2C9 *1/*2	7 (15)	Intermediate metabolizer	7 (15)	1 (40)	6 (48)
CYP2C9 *2/*3	12 (26)	Poor metabolizer	12 (26)	12 (22)	0 (0)

**Table 5:** Genotype and phenotype distribution for CYP2C19.

Genotype	n (%)	Phenotype	n (%)	Men n (%)	Women n (%)
CYP2C19 *1/*1	43 (48)	Normal (rapid) metabolizer	43 (48)	23 (40)	20 (59)
CYP2C19 *1/*2	11 (12)	Intermediate metabolizer	20 (22)	8 (14)	3 (9)
CYP2C19 *2/*17	9 (10)			5 (9)	4 (12
CYP2C19 *2/*2	4 (4)	Slow metabolizer	6 (6)	2 (3)	2 (6)
CYP2C19 *2/*3	2 (2)			2 (3)	0 (0)
CYP2C19 *1/*17	15 (17)	Ultrarapid metabolizer	21 (24)	12 (21)	5 (15)
CYP2C19 *17/*17	6 (7)			6 (10)	0 (0)

**Table 6:** Genotype and phenotype distribution for CYP2D6.

Genotype	n (%)	Phenotype	n (%)	Men n (%)	Women n (%)
CYP2D6 *1/*1	3 (17)	Normal (rapid) metabolizer	7 (40)	1 (100)	2 (12)
CYP2D6 *1/*33	1 (6)			0	1 (6)
CYP2D6 *1/*35	3 (17)			0	3 (18)
CYP2D6 *35/*35	1 (6)			0	1 (6)
CYP2D6 *1/*5	2 (11)	Intermediate metabolizer	7 (39)	0	2 (12)
CYP2D6 *1/*5	5 (28)			0	5 (29)
CYP2D6 *4/*15	2 (11)	Slow metabolizer	3 (17)	0	2 (12)
CYP2D6 *5/*40	1 (6)			0	1 (6)

**Table 7:** Genotype and phenotype distribution for TPMT.

Genotype	n (%)	Phenotype	n (%)	Men n (%)	Women n (%)
TPMT *1	61 (88)	Normal (rapid) metabolizer	61 (88)	24 (69)	20 (59)
TPMT *3A	4 (6)	Slow metabolizer	8 (12)	6 (17)	6 (18)
TPMT *3B	4 (6)			5 (14)	8 (24)

TPMT—thiopurine S-methyltransferase.

**Table 8:** Detection of mutant variant rs6025 and genotype distribution for factor V Leiden.

Detection	n (%)	Genotype	n (%)	Men n (%)	Women n (%)
Negative	26,289 (84)	Normal (no rs6025 mutation)	26,289 (84)	4975 (79)	21,314 (86)
Positive	4941 (16)	Heterozygous rs6025 mutation	3516 (11)	1067 (17)	2449 (9)
		Homozygous rs6025 mutation	1425 (5)	265 (4)	1160 (5)

## Data Availability

Data is available to researchers on demand.
